# Genetics and Genomic Regions Affecting Response to Newcastle Disease Virus Infection under Heat Stress in Layer Chickens

**DOI:** 10.3390/genes10010061

**Published:** 2019-01-18

**Authors:** Perot Saelao, Ying Wang, Ganrea Chanthavixay, Rodrigo A. Gallardo, Anna Wolc, Jack C. M. Dekkers, Susan J. Lamont, Terra Kelly, Huaijun Zhou

**Affiliations:** 1 Integrative Genetics and Genomics Graduate Group, University of California, Davis, CA 95616, USA; psaelao@ucdavis.edu (P.S.); kchantha@ucdavis.edu (G.C.); 2 Genomics to Improve Poultry Innovation Lab, University of California, Davis, CA 95616, USA; ucywang@ucdavis.edu (Y.W.); trkelly@ucdavis.edu (T.K.); 3 Department of Animal Science, University of California, Davis, CA 95616, USA; 4 School of Veterinary Medicine, University of California, Davis, CA 95616, USA; ragallardo@ucdavis.edu; 5 Department of Animal Science, Iowa State University, Ames, IA 50011, USA; awolc@iastate.edu (A.W.); jdekkers@iastate.edu (J.C.M.D.); sjlamont@iastate.edu (S.J.L.); 6 Hy-Line International, Dallas Center, IA 50063, USA

**Keywords:** NDV, GWAS, heat stress, chicken, QTL

## Abstract

Newcastle disease virus (NDV) is a highly contagious avian pathogen that poses a tremendous threat to poultry producers in endemic zones due to its epidemic potential. To investigate host genetic resistance to NDV while under the effects of heat stress, a genome-wide association study (GWAS) was performed on Hy-Line Brown layer chickens that were challenged with NDV while under high ambient temperature to identify regions associated with host viral titer, circulating anti-NDV antibody titer, and body weight change. A single nucleotide polymorphism (SNP) on chromosome 1 was associated with viral titer at two days post-infection (dpi), while 30 SNPs spanning a quantitative trait loci (QTL) on chromosome 24 were associated with viral titer at 6 dpi. Immune related genes, such as CAMK1d and CCDC3 on chromosome 1, associated with viral titer at 2 dpi, and TIRAP, ETS1, and KIRREL3, associated with viral titer at 6 dpi, were located in two QTL regions for viral titer that were identified in this study. This study identified genomic regions and candidate genes that are associated with response to NDV during heat stress in Hy-Line Brown layer chickens. Regions identified for viral titer on chromosome 1 and 24, at 2 and 6 dpi, respectively, included several genes that have key roles in regulating the immune response.

## 1. Introduction

Newcastle disease virus (NDV) is a pathogen that is known to infect over 200 different species of birds, including chickens [1]. The most virulent forms of NDV have historically impacted poultry producers worldwide. From 2006 to 2009, Newcastle disease was the most widespread animal disease in terms of total number of countries affected [2]. NDV, a member of the family *Paramyxoviridae*, is a single-stranded, negative-sense RNA virus with several different strains that is defined based on their pathogenicity [1]. Typically, commercial chickens are vaccinated during their production cycle to prevent NDV infection. However, infections can still occur in vaccinated birds, resulting in respiratory disease and a significant drop in egg production, among other clinical signs [3,4]. Although vaccination has been critical in reducing the number of NDV outbreaks around the world, genetic selection on existent genetic variation for resistance can further aid in reducing the prevalence of this pathogen in areas in which it is difficult to maintain protective vaccination levels [5]. Genetic resistance to NDV has been observed within various lines and breeds of chickens [6,7,8,9], supporting the potential of utilizing inherent genetic resistance to NDV.

In addition to the devastating potential of NDV for poultry production, environmental factors, such as heat stress, exacerbate losses in poultry production. Heat stress alone is estimated to result in over $165 million U.S. dollars annually in global poultry losses [10]. Physiological systems are significantly altered during heat stress, with negative impacts on body weight and immunity [11]. Bartlett and Smith observed that heat stress reduced the total level of antibodies during primary and secondary immune responses in chickens [12]. Studies in cell lines that investigated the stimulation of the immune response through lipopolysaccharide treatment during heat stress found that heat shock proteins may be one of the primary drivers of the immune response during times of abiotic stress [13]. Studies on the seasonality of NDV outbreaks have noted a higher incidence during periods of climatic stress. Reports of NDV outbreaks in Uganda, Nepal, and Zambia have noted the highest incidences to occur during the hot dry season and the hot humid season [14,15,16], which highlights the need for further studies to understand the immune response to NDV infection under environmental stress.

Previous studies have revealed candidate loci associated with differences in response to NDV infection on several chromosomes in broiler chickens and in intercrossed lines [17,18,19]. However, few studies have attempted to identify disease resistance associated loci while under the effects of abiotic stress. The aim of this study was to use estimate genetic parameters and use genome-wide association studies (GWAS) to identify quantitative trait loci (QTLs) associated with viral load, antibody response, and body weight change in Hy-Line Brown chickens using a high-density single nucleotide polymorphism (SNP) array.

## 2. Materials and Methods

### 2.1. Experimental Population

Hy-Line International Brown laying chickens (Hy-Line Brown, Hy-Line International, West Des Moines, IA, USA) were utilized in this experiment. Three replicate trials of mixed-sex chicks (180 birds per trial) were conducted using a similar protocol as described by Wang et al. [20]. In brief, on the day of hatching, chicks were transported from Dallas Center, Iowa to a Bio Safety Level-2 animal facility at the University of California, Davis, and housed in temperature and humidity-controlled chambers. Twenty individuals per trial, randomly selected from 60 dams, were housed in separate chambers and used as a control group. From day 1, both groups were reared at 35 °C and 60% humidity. Up to day 13, the temperature for both groups was then gradually decreased to 29.4 °C. At 14 days of age, the treatment group was exposed to 35 °C and maintained at this temperature until the conclusion of the trial. The control group was maintained at 25 °C. On day 21, the heat-treated birds were inoculated with 200 μL 10^7^ EID_50_ of the La Sota strain of NDV through both ocular and nasal passages. The treated group refers to birds that were under the effects of both heat stress and NDV infection, while non-treated birds were mock-inoculated with 200 μL of 1X phosphate-buffered saline (PBS) and under non-heat stressed conditions. The experimental trial was performed according to guidelines approved by the Institutional Animal Care and Use Committee at the University of California, Davis (IACUC #17853).

### 2.2. Phenotypic Measurements

Chicken lachrymal fluid was collected at 2 and 6 dpi on treated birds. Viral RNA was extracted using the MagMAX-96 viral RNA isolation kit (Life Technologies, Carlsbad, CA, USA, Cat#AMB18365). Quantification of viral titer was conducted using quantitative RT-PCR (qRT-PCR) with the TaqMan Newcastle Disease Virus Real-Time PCR kit (Life Technologies Cat#44006874) and measured on the ABI 7500 fast Real-Time PCR system (Thermofisher Cat#4351107, Carlsbad, CA, USA). A standard curve was generated from a log copy number dilution of 10^5^ to 10^2^ EID_50_ of the virus and used to calculate the viral titer of the sample. Viral clearance was measured as the difference between viral titer at 2 and 6 dpi, divided by the viral titer at 2 dpi. 

Anti-NDV antibodies were measured in serum collected at 10 dpi using the IDEXX NDV ELISA kit for chickens (IDEXX Laboratories, Westbrook, ME, USA, Cat#99-09263). The sample to positive ratio (S/P) was calculated as the average absorbance of each sample divided by the recorded measurement of the provided kit control. Body weight measurements (g) were taken at day 1, 13, 14, 21, 27, and 30.

### 2.3. Genotyping

Genomic DNA was extracted from blood preserved on Whatman FTA cards (GE Healthcare, Chicago, IL, USA, CAT#WB120401). A total of 526 individuals that met concentration and DNA quality requirements were genotyped on the Axiom 600 K Genome-Wide Chicken Array Kit (Affymetrix, Santa Clara, CA, USA, CAT#902148) by Geneseek (Lincoln, NE, USA). SNPs were then filtered for a call rate greater than 95% and minor allele frequency of greater than 0.01. After quality filtering, 304,500 SNPs were used in the downstream analysis from 526 individuals. SNP positions were mapped to the galGal 5 reference genome.

### 2.4. Data Analysis

Statistical significance of differences in body weight measurements between the treated and untreated groups were done using a Student’s *t*-test in R [21]. ASReml 4 was used to estimate heritability and correlations on the treated group only [22]. The animal model utilized was:

$$Y_{ijkl}=µ+S_{i}+Rep{\left(C\right)}_{jk}+A_{l}+e_{ijkl}$$

where *Y* is the dependent variable of the analyzed trait, μ is the mean of the individual trait, sex (*S*), and chamber nested within replicate (*Rep*(*C*)) were fitted as fixed effects. Random effects included animal genetic effects (*A*), using a genomic relationship matrix based on Reference [23], and residuals (*e*). For viral titer measurements at 2 and 6 dpi, dam anti-NDV antibody level was added as a covariate. Heritability was estimated as the ratio of the animal variance to phenotypic variance and phenotypic and genetic correlations were estimated using corresponding bivariate analyses.

Genome-wide association analysis was performed using the R package GenABEL [24], using the function “polygenic_hglm” to implement a polygenic model based on a hierarchical generalized linear model, fitting the same fixed effects as the ones used for genetic parameter estimation. We utilized methods designed by Aulchenko et al. to generate the genomic relationship matrix and associations [24].

To establish significance thresholds, the number of independent tests was determined as the number of principal components that accounted for 95% of the variance in SNP genotypes, as described by Waide et al. [25,26]. The number of independent tests was used in a Bonferroni correction to determine the 20% and 10% genome-wide significance levels. A total of 49,138 principal components were determined to account for 95% of the variance between SNPs. Using the adjusted Bonferroni cutoff, the *p*-values for the 10 and 20% genome-wide significance thresholds were 2.04 × 10^−6^ and 4.07 × 10^−6^. SNPs were then filtered using the 10 and 20% genome-wide significance thresholds and their Affymetrix SNP IDs were converted into SNP ID’s using National Center for Biotechnology Information’s dbSNP database [27]. SNPs were then associated with genes using the Ensembl Variant Effect Predictor web tool [28] using a 1 Mb window. Linkage disequilibrium based on *r*^2^ for all pairs of SNPs were estimated using the linkage disequilibrium function in PLINK [29] with a 1 Mb window size. Body weight gain was calculated as the difference in body weight from day 1 to the corresponding time point in grams.

## 3. Results

### 3.1. Quantification of NDV Titers and Anti-NDV Antibody Levels in Hy-Line Brown Layer Chicks

**Figure 1:**
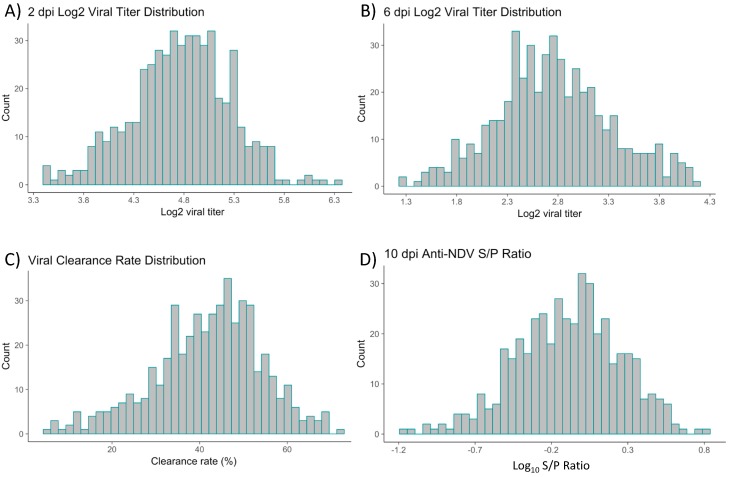
Viral titer, viral clearance, and anti-NDV antibody quantification of Hy-Line Brown birds by days post infection (dpi). (**A**) Log_10_ viral titer distribution at 2 dpi and (**B**) Log_10_ viral titer distribution at 6 dpi. (**C**) Viral clearance rate measured as the percent change in viral titer from 2 to 6 dpi. (**D**) Anti-NDV antibody measured using ELISA and depicted as the log_10_ of the least-square mean of the S/P ratio at 10 dpi. NDV: Newcastle disease virus.

Following infection at 21 days of age, Newcastle disease virus was isolated from lachrymal fluid and quantified using qRT-PCR on 526 commercial layer chickens at both 2 and 6 days post-infection (dpi, Figure 1). The mean ± standard error of the log_10_ viral copy number was 4.77 ± 0.022 and 2.76 ± 0.026 at 2 and 6 dpi, respectively (Table 1). Viral clearance rate, calculated as the difference in viral copy number from 2 to 6 dpi divided by viral copy number at 2 dpi, was also calculated, with a mean viral clearance rate of 41.7% ± 0.037 (Figure 1). Antibody response to NDV was measured at 10 dpi using an enzyme linked immunosorbent assay (ELISA). The mean anti-NDV log_10_ (sample to positive ratio) of the population was −0.10 ± 0.016, and 1.04 ± 0.037 on the untransformed scale (Figure 1). Maternally inherited anti-NDV antibody titers were measured at day 20 of age and were not detected in any individuals.

### 3.2. Body Weight Gain Across Time Points

**Figure 2:**
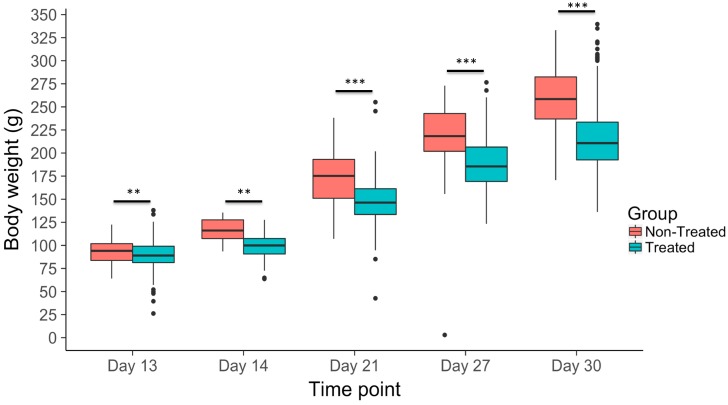
Body weight change from day 1 to day 13–30 of treated (NDV infected and heat stressed) and non-treated birds (non-infected at ambient temperature). Although minor body weight differences existed prior to heat treatment at on day 13, average body weights of treated and non-treated birds deviated drastically after heat treatment and all time points following NDV infection (day 21–30). * *p*-value < 0.05, ** *p*-value < 0.01, *** *p*-value < 0.0001.

Body weight gain (BWG) of both treated (either heat or combination of heat and NDV infection) and non-treated individuals was measured at 14, 21, 27, and 30 days of age in order to assess the effects of heat treatment and both heat treatment and NDV infection on growth rate (Figure 2, Table 1). Significant differences (*p*-value < 0.05) in BWG were observed between treated and non-treated individuals across all recorded time points (days 14, 21, 27, and 30). However, prior to both heat and NDV treatment, differences in BWG existed between the two groups at day 13, although the two groups of birds were randomly selected from each dam family and assigned to treatments (Table 1).

### 3.3. Genetic Parameters

ASReml data analysis software was used to estimate the heritability for all traits (Table 1). Viral titer at 2 and 6 dpi had estimated heritabilities of 0.17 ± 0.096 and 0.11 ± 0.081, respectively. The estimate of heritability for anti-NDV antibody was 0.039. NDV clearance rate also had a very low heritability estimate (not significantly different from 0). Body weight gain across the various time points had heritability estimates that ranged from 0.13 to 0.28. Genetic and phenotypic correlations were estimated using bivariate analyses in ASReml. Phenotypically, viral titer at 2 and 6 dpi were positively correlated. Both of these traits were also positively correlated with anti-NDV antibody levels. Genetically, viral titer at 2 dpi was positively correlated with both 6 dpi viral titer and anti-NDV antibody level. However, 6 dpi viral titer had a negative genetic correlation with anti-NDV antibody levels (Table 2).

### 3.4. Genome-Wide Association Analysis

**Figure 3:**
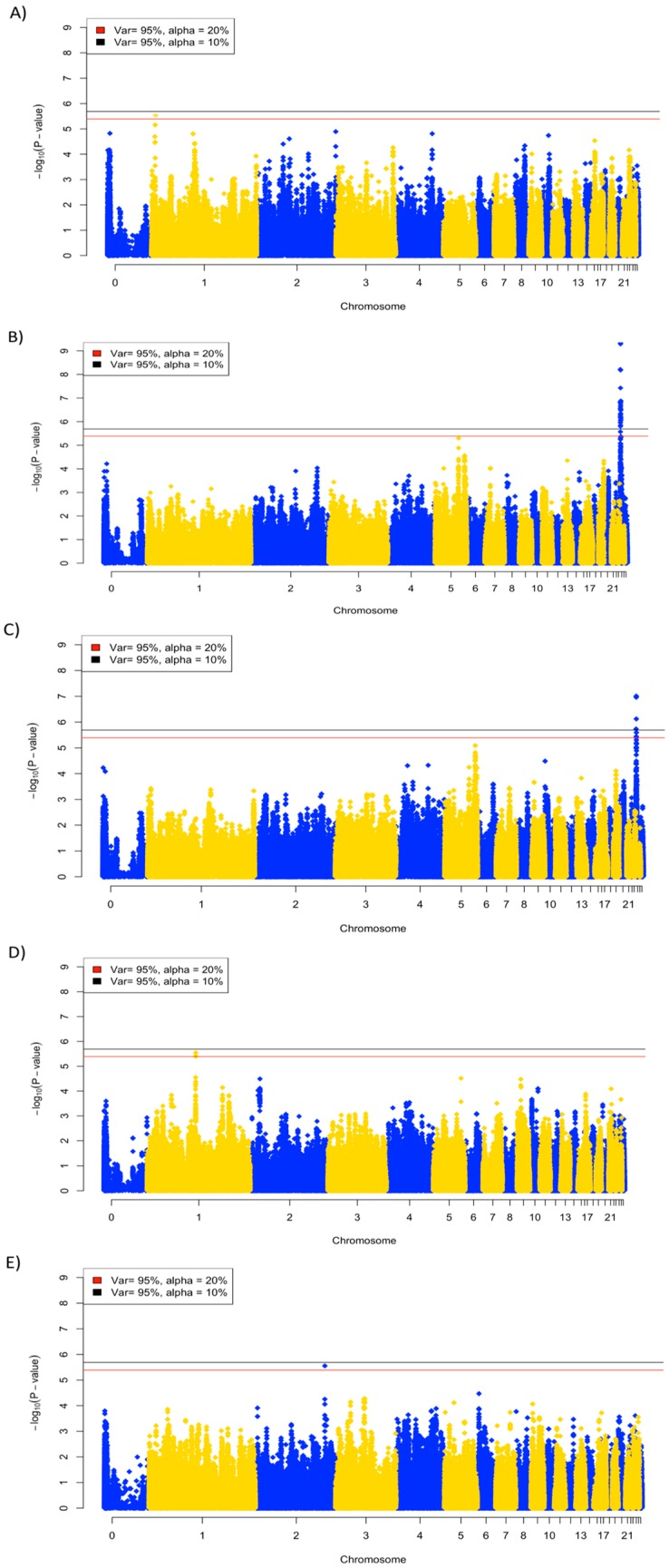
Manhattan plots of the genome-wide association analysis of traits, with suggestive SNPs at the 10 (black) and 20% (red) genome-wide significance: (**A**) viral titer at 2 dpi; (**B**) viral titer at 6 dpi; (**C**) viral clearance; (**D**) log_10_ anti-NDV antibody S/P ratio at 10 dpi, and (**E**) day 13 body weight change.

A total of 526 individuals and 304,500 SNPs passed quality filtering and were utilized in the GWAS analysis. An adjusted Bonferroni cutoff for 10% (*p*-value = 2.035 × 10^−6^) and 20% (*p*-value = 4.0701 × 10^−6^) genome-wide significance, based on the number of independent tests that accounted for 95% of the variation in SNP genotypes, was used to identify suggestive QTL. GWAS was performed for each trait and results are presented as Manhattan plots (Figure 3A–F). Of the nine traits, five had SNPs at 20% genome-wide significance, while two had significant SNPs at 10% genome-wide significance (Table 3). SNP rs316767446 (*p*-value = 3.92 × 10^−6^) on chromosome 1 was the only suggestive association found for the 2 dpi viral titer. The viral titer at 6 dpi had the strongest signal detected, with 30 and 29 suggestive SNPs at 20% and 10% significance, respectively. These SNPs were all located on chromosome 24, in a region from 0.4 to 1.4 Mb (Additional File 1). Viral clearance rate had seven suggestive SNPs identified on chromosome 24, approximately in the same region as identified at 6 dpi. The GWAS for anti-NDV antibody identified three SNPs (rs1390558, rs31296314, and rs3147209) on chromosome 1 below the 20% significance cutoff. BWG had only one associated SNP identified at 20% genome-wide significance, on chromosome 2 at day 13.

The average linkage disequilibrium (LD) in the Hy-Line Brown layer chickens ranged from 0.55 to 0.58 (*r*^2^) at 0.1 to 1.0 Mb distance. Other measures in the Hy-Line layer lines by Abasht et al. [30] reported average LD ranging from 0.88 to 0.92 (*r*^2^) over these same distances. Based on this, gene annotation in a 1 Mb region around each significant SNP was searched to identify functionally relevant genes that may be responsible for the SNP associations for each trait. A table containing a summary of the known functions of the genes identified in this study can be found in the Supplementary Information (Additional File 2). For the QTL centered around the only suggestive SNP, rs316767446, associated with the 2 dpi viral titer, 17 genes were identified within its boundary (Table 4). This region includes immune related genes such as *CDC123*, *CAMK1d*, and *CCDC3*. SNPs associated with viral load at 6 dpi identified a 1 Mb QTL on chromosome 24, with 43 genes located within this region, including candidate genes *ST3GAL4*, *TIRAP*, *FLI1*, and *ETS1*, which are involved in functions related to the inflammatory and innate immune response (*ST3GAL4* and *TIRAP*) [31,32], B cell proliferation *(FLI1*), and the expression of cytokine and chemokine genes (*ETS1*) [33,34]. *ETS1* was also located within the QTL region identified for viral clearance. QTL analysis for anti-NDV antibody levels revealed two genes, *POU1F1* and *CHMP2B*, that were in close proximity to the significant SNPs. No genes were found within the 1 Mb region for SNPs identified for BWG at day 13.

## 4. Discussion

To the best of our knowledge, this study provides the first estimates of heritability for NDV viral titer in lachrymal fluid while under the effects of heat stress. Overall, heritability estimates were lower than those reported in investigations into other populations of chickens of similar phenotypic traits [17,35,36], such as body weight and anti-NDV antibody response. However, many of these studies estimated heritability under non-stressful environmental conditions. Estimates for the same line of birds exposed only to NDV infection observed heritabilities of viral titer at 2 and 6 dpi, and for anti-NDV antibody titer at 10 dpi of 0.32, 0.18, and 0.24, respectively [37]. Environmental factors can impact heritabilities, where genes involved in specific traits may respond differently according to environmental factors [38].

This study revealed suggestive QTL for NDV viral titers at 2 and 6 dpi on chromosomes 1 and 24, respectively. Although the significance thresholds used in this study deviate from the traditional cutoffs typically used, the results generate candidate regions for functional association between SNPs and phenotype. QTL for anti-NDV antibody levels were previously reported on chromosome 1 [17] but these QTL did not co-localize with the suggestive QTL we identified in this study. Of particular interest are three immune-related genes (*CDC123*, *CAMK1d,* and *CCDC3*) located within the 1 Mb QTL region identified on chromosome 1 centered around the suggestive SNP associated with the viral titer 2 dpi. *CDC123* is a gene that is highly expressed in leukocytes and plays a role in eIF2 regulation and internal host-pathogen interaction [39]. Unpublished data from our group also found *CDC123* to be significantly up-regulated in NDV infected and heat stressed Fayoumi chickens compared to non-treated birds. *CAMK1d* has been associated with regulation of granulocyte function and neutrophil activation during influenza virus stimulation, while *CCDC3* negatively regulates the TNF-α-induced pro-inflammatory response via inhibition of TNF-α-induced NF-κB activation [40,41]. In addition, another study suggested that alternatively spliced variants of *CAMK1d* may protect against influenza infection [41]. The *CAMK1d* gene is also a target for microRNA silencing by viruses and, thus, inhibition of this gene can facilitate viral invasion into the host cell [41]. Collectively, these three genes and the genetic variants identified in this study warrant further investigation to elucidate their roles in regulating the host’s response to NDV infection.

The QTL affecting the viral titer at 6 dpi had the largest number of SNPs identified in this study. The approximately 1 Mb region on chromosome 24 contained 29 and 30 SNPs that were significant at the 10 and 20% genome-wide significance levels, respectively, and encompassed seventeen genes. Several of these genes, including *ETS1*, *TIRAP*, *FLI1*, *ST3GAL4*, and *KIRREL3*, provide potential candidates that may be associated with a viral titer at 6 dpi. *ETS1* had five significant SNPs located within 100 kb of it. *ETS1* is a transcription factor that functions as a regulator of cytokine and chemokine gene expression [33] and was also found to be up-regulated and differentially expressed in Fayoumi birds during NDV infection and heat stress in our unpublished results. ETS1 may be a critically important gene at this early stage of activating the adaptive immune response that chickens rely upon when infected by NDV. Other immune response genes in this QTL region, such as *TIRAP*, *FLI1*, and *ST3GAL4*, could be critical in terms of the broader response to the virus at 6 dpi. *TIRAP* is a Toll-interleukin 1 receptor adaptor protein that is part of the microbial pathogen recognition system of Toll-like receptors and functions in pathogen recognition within the host [39]. TIRAP codes for proteins that activate *TLR4* signaling and initiates genes such as *NF-κB*, *MAPK1*, *MAPK3*, and *JNK* for cytokine secretion and inflammatory response. *TIRAP* plays a larger role in modulating the early innate immune response and *TLR4*’s antiviral response through viral envelope glycoprotein detection [42]. *FLI1* is a DNA-binding protein with a similar binding domain to *ETS1*. Reduced expression of *FLI1* in transgenic mice was reported to cause deficiencies in proliferative response of B cells to mitogens [34]. *ST3GAL4* has been demonstrated to be the major sialytransferase involved in producing selectin ligands, which promote leukocyte trafficking and mobilization [43]. These candidate genes may all contribute to transition of host immune response from innate to adaptive immunity to NDV infection. The *KIRREL3* gene had the largest number of significant SNPs (7) identified within a gene in this study. *KIRREL3* is involved in developmental anomalies and defective *KIRREL3* expression has been associated with failures in immune response [44]. Several SNPs located near *KIRREL3* (rs318146300, rs316424273, rs3164202764) were also significantly associated with the viral clearance QTL that was identified in this study, which provides further evidence of the potential role this gene may play in regulation of the immune response to NDV while under heat stress.

Suggestive SNPs associated with anti-NDV antibody levels identified in this study overlapped with regions on chromosome 1 that were identified in a previous study [17]. The *POU1F1* and *CHMP2B* genes are located within the 1 Mb QTL window centered around the suggestive SNPs found on chromosome 1. The *CHMP2B* gene was found to be differentially expressed between NDV infected and heat stressed, and non-treated Fayoumi chickens in unpublished results from our group. This suggests that *CHMP2B* is related to NDV resistance, as Fayoumi birds have been previously shown to be relatively more resistant to NDV [7]. In addition, a parallel study on the same line of birds conducted at Iowa State University to identify QTL for the host response to NDV infection without heat stress identified QTL on chromosome 21 associated with anti-NDV antibody level and on chromosome 4 for a viral titer at 6 dpi [37]. Interestingly, these two regions associated with NDV traits were not observed within this study when heat stress was added as an additional factor. Despite similar estimates of genetic parameters between these two studies, QTL regions identified did not appear to be shared. Although limited power to detect QTL due to environmental and location may be significantly contributing factors, the lack of overlap in results between the two parallel studies may provide insight into the impact of heat stress on response to NDV infection. Previous studies on genetic markers associated with anti-NDV antibodies in other chicken lines identified QTL on chromosomes 1, 3, 4, 5, 9, 13, 16, and 22 [17,18,19]. Several possible explanations for why QTLs identified in other studies were not discovered in the current study: (1) genetic composition of the experimental populations used, as previous studies primarily used broiler chickens or a combination of laying lines, each of which may be displaying line specific or breed specific QTLs for anti-NDV antibody response; (2) the number of SNPs or marker density used, where the current study utilized a 600k SNP panel compared to a 50k SNP panel or microsatellite markers in previous studies; (3) time post-vaccination and the dose of NDV applied; (4) the use of heat stress as an additional treatment; and (5) the limited power of most QTL mapping studies. Few studies have conducted GWAS under the effect of both heat stress and pathogen infection and, thus, many of the QTL previously identified may be missed due to the combination of impacting factors. This highlights the importance of considering the resource population utilized when attempting to translate results to direct application, and the impact environmental factors have on QTLs effects.

NDV infection and/or heat stress appeared to significantly reduce body weight gain of treated individuals compared to the non-treated individuals across all measured time points. The chicks used in the non-treated and treated groups were randomly selected from within dam families in order to maximize the representation of all sixty dams within the population. However, this random selection within each dam group unexpectedly resulted in significant differences in BWG between the treated and non-treated groups prior to the treatment at day 13. Although the differences in BWG between the two groups continually increased with time of treatment, as expected (Figure 2), this potentially confounding effect must be considered when evaluating the direct effect of treatment on growth rate in this study.

## 5. Conclusions

Estimates of heritabilities and the QTL regions that may be putatively controlling traits related to NDV infection during heat stress that were identified in this study provide insights into the genetic control of these traits and provide genetic markers that can be used for selective breeding programs to enhance NDV resistance in chickens in hot environments. Specifically, suggestive QTL regions found on chromosomes 1 and 24 revealed several candidate genes (*KIRREL3, TIRAP, ETS1, CCDC3,* and *CAMK1d*) that may play important roles in the chicken’s response to NDV during heat stress. The variants identified in this study warrant further investigation to understand the underlying molecular mechanisms of action. These results further the overall goal of the Feed the Future Innovation Lab to develop genetically enhanced, resilient poultry and thus mitigate the impacts of NDV and climate change across the globe.

## Figures and Tables

**Table 1:** Trait means and estimates of heritability with standard errors in the heat-stressed and NDV infected Hy-Line Brown population.

Trait	Mean ± SE	h^2^ ± SE
Viral titer, 2 dpi	4.77 ± 0.022	0.17 ± 0.096
Viral titer, 6 dpi	2.76 ± 0.026	0.11 ± 0.080
Anti-NDV antibody	−0.10 ± 0.037	0.039 ± 0.063
Viral clearance	41.74 ± 0.57	
Body weight change, day 13	89.39 ± 0.58	0.13 ± 0.076
Body weight change, day 14	99.28 ± 0.98	0.18 ± 0.17
Body weight change, day 21	147.54 ± 0.93	0.26 ± 0.11
Body weight change, day 27	190.69 ± 1.53	0.22 ± 0.11
Body weight change, day 30	215.89 ± 1.54	0.28 ± 0.094

**Table 2:** Estimates of phenotypic (above diagonal) and genetic correlations (below diagonal) of NDV related traits.

	Viral Titer, 2 Dpi	Viral Titer, 6 Dpi	Anti-NDV Antibody
Viral titer, 2 dpi		0.14 ± 0.048	0.11 ± 0.05
Viral titer, 6 dpi	0.51 ± 0.40		0.0199 ± 0.0493
Anti-NDV antibody	0.35 ± 0.57	−0.33 ± 0.79	

**Table 3:** Number of significant SNPs identified at the 10 and 20% genome-wide significance levels across all traits.

Trait	Number of Significant SNPs	
	10% Significance	20% Significance
Viral titer, 2 dpi	0	1
Viral titer, 6 dpi	29	30
Anti-NDV antibody	0	3
Viral clearance	4	7
Body weight change, day 13	0	1
Body weight change, day 14	0	0
Body weight change, day 21	0	0
Body weight change, day 27	0	0
Body weight change, day 30	0	0

**Table 4:** QTL region positions and genes located within 1 Mb of the suggestively significant SNPs for the evaluated traits.

Trait	Number of SNPs	Chr: Mb	Genes
Viral titer, 2 dpi	1	1: 6.1–7.1	*CDC123, CAMK1D, CCDC3, DHTKD1, BEND7, ECHDC3, PROSER2, OPTN, NUDT5, SEPHS1, gga-mir-1460, FRMD4A, MCM10, UPF2, USP6NL, PHYH, SEC61A2*
Viral titer, 6 dpi	30	24: 0.1–1.9	*TIRAP, SRPRA, KIRREL3, ST3GAL4, EI24, FAM118B, DCPS, CDON, STT3A, CHEK1, FOXRED1, ETS1, FL11, KCNJ1, KCNJ5, 5S_rRNA, ARHGAP32, PANX3, PUS3, NRGN, RPUSD4, C2CD2L, VPS11, SLC37A2, MSANTD2, SIK2, DPAGT1, HMBS, PKNOX2, VSIG10L2, FEZ1, HYLS1, ESAM, HEPACAM, ROBO3, RF00001, BARX2, TMEM45B, APLP2, NFRKB, ST14, ZBTB44, ADAMTS8, ADAMTS15*
Viral clearance	7	24: 0.1–1.1	*TIRAP, SRPRA, KIRREL3, ST3GAL4, EI24, FAM118B, DCPS, CDON, STT3A, CHEK1, FOXRED1, ETS1, FL11, KCNJ1, KCNJ5, 5S_rRNA, ARHGAP32*
Body weight change, day 13	1	2: 128.8–129.8	

## Supplementary Materials

The following are available online at http://www.mdpi.com/2073-4425/10/1/61/s1. Additional File 1: Suggestively significant SNPs identified in this study and the associated SNP ID information, Additional File 2: Summary of the known function of genes identified within this study.
